# Nutritional Potential and Toxicological Evaluation of *Tetraselmis* sp. CTP4 Microalgal Biomass Produced in Industrial Photobioreactors

**DOI:** 10.3390/molecules24173192

**Published:** 2019-09-03

**Authors:** Hugo Pereira, Joana Silva, Tamára Santos, Katkam N. Gangadhar, Ana Raposo, Cláudia Nunes, Manuel A. Coimbra, Luísa Gouveia, Luísa Barreira, João Varela

**Affiliations:** 1 CCMAR—Centre of Marine Sciences, University of Algarve, Gambelas, 8005-139 Faro, Portugal; 2 CMP—Cimentos Maceira e Pataias, ALGAFARM - Unidade de Produção de Microalgas, 2445-411 Pataias, Portugal; 3 LEPABE—Department of Chemical Engineering, Faculty of Engineering, University of Porto, 4200-465 Porto, Portugal; 4 CICECO—Aveiro Institute of Materials and Department of Chemistry, University of Aveiro, 3810-193 Aveiro, Portugal; 5 QOPNA and Department of Chemistry, University of Aveiro, 3810-193 Aveiro, Portugal; 6 LNEG—Laboratório Nacional de Energia e Geologia, I.P./Bioenergy Unit, Estrada do Paço do Lumiar 22, 1649-038 Lisbon, Portugal

**Keywords:** microalgae, biochemical composition, *Tetraselmis* sp. CTP4, pigments, vitamins, antioxidants, toxicological evaluation

## Abstract

Commercial production of microalgal biomass for food and feed is a recent worldwide trend. Although it is common to publish nutritional data for microalgae grown at the lab-scale, data about industrial strains cultivated in an industrial setting are scarce in the literature. Thus, here we present the nutritional composition and a microbiological and toxicological evaluation of *Tetraselmis* sp. CTP4 biomass, cultivated in 100-m^3^ photobioreactors at an industrial production facility (AlgaFarm). This microalga contained high amounts of protein (31.2 g/100 g), dietary fibres (24.6 g/100 g), digestible carbohydrates (18.1 g/100 g) and ashes (15.2 g/100 g), but low lipid content (7.04 g/100 g). The biomass displayed a balanced amount of essential amino acids, *n*-3 polyunsaturated fatty acids, and starch-like polysaccharides. Significant levels of chlorophyll (3.5 g/100 g), carotenoids (0.61 g/100 g), and vitamins (e.g., 79.2 mg ascorbic acid /100 g) were also found in the biomass. Conversely, pathogenic bacteria, heavy metals, cyanotoxins, mycotoxins, polycyclic aromatic hydrocarbons, and pesticides were absent. The biomass showed moderate antioxidant activity in several in vitro assays. Taken together, as the biomass produced has a balanced biochemical composition of macronutrients and (pro-)vitamins, lacking any toxic contaminants, these results suggest that this strain can be used for nutritional applications.

## 1. Introduction

Microalgae are a polyphyletic group of highly biodiverse photosynthetic unicellular or colonial microorganisms occurring in almost every known habitat, ranging from oceans to deserts. These microorganisms are currently considered to be one of the solutions to meet the high demand for food and feed caused by the expected growth of the human population in the forthcoming decades [1]. Moreover, microalgae usually couple a balanced nutritional profile with the presence of bioactive molecules. This combination can be used to implement new functional foods by using microalgal biomass alone or as a functional ingredient to fortify/supplement traditional food products, so that their basic nutritional value is improved, enhancing the health benefits of food and feed [2,3,4,5]. The use of microalgae as functional foods has been proposed to be beneficial to healthy individuals but also as a way of decreasing the risk of illness [4]. In this context, regular consumption of functional foods is expected to counteract risk factors that are known to be associated with different forms of non-communicative chronic conditions, such as cancer and cardiovascular and neurodegenerative disorders [6,7]. Several microalgal strains are rich in *n*-3 fatty acids (e.g., eicosapentaenoic (EPA) and docosahexaenoic (DHA) acids), phytosterols, polyphenols, vitamins, phycobiliproteins, and carotenoids (e.g., lutein, astaxanthin, and β-carotene), among others [8,9,10]. These metabolites are commonly found in microalgal biomass and are highly relevant, since they are known to display a wide range of biological activities. For example, it has been reported that microalgal biomass contains molecules with antioxidant, metal-chelating, antidiabetic, antibiotic, antifungal, antiviral, anti-inflammatory, anticancer, and neuroprotective properties [11,12,13,14,15]. Furthermore, *n*-3 fatty acids and carotenoids are frequently recognized as molecules that could prevent medical conditions, such as cardiovascular and autoimmune diseases [16]. Accordingly, the use of whole microalgal biomass or extracts prepared therefrom hold high potential for biomedical applications, including the manufacture of nutraceuticals and the development of promising leads for pharmaceutical drugs [6,17].

Commercial production of microalgal biomass has mainly been carried out for the animal and human nutrition markets. Their high protein content and the presence of essential fatty acids and vitamins were essential for their adoption as sources of food and feed [8]. In aquaculture, for example, they have an important role during the first stages of fish larvae rearing and bivalve cultivation. Regarding human nutrition, from the beginning of the 1960s, microalgae, such as *Arthrospira* (formerly known as *Spirulina*) and *Chlorella*, have been commercially produced either for direct consumption or as food supplements [8]. In fact, the utilization of microalgae is a growing trend in Europe, with several authors claiming that microalgal biomass can be a “functional/super food” [18]. Unfortunately, only a very limited number of microalgal species have been classified as food ingredients by the European Food Safety Agency (EFSA). For the introduction of a novel species in the Europe Union (EU) food market, a Novel Food Dossier must be submitted, and the “novel food” status obtained. Dried *Tetraselmis chui* is one of those examples, having achieved the novel food status in 2014.

The *Tetraselmis* genus is known to hold promising nutritional properties, associated to different biological activities, including antioxidant, metal-chelating, neuroprotective, cell repairing, and cytotoxic activities [6,17,19]. Although different reports detail the relevance of this genus as feedstock for carbohydrates [20], protein [21], and lipids [22], it has become apparent that the biochemical composition among strains grown in specific cultivation systems can change considerably, in particular when comparing results obtained at lab- and industrial-scales. As *Tetraselmis* sp. CTP4 is a recently isolated industrial strain displaying high growth rates under stressful conditions and robustness against potential predators and competitors [22,23], showing strong potential for bioremediation [24] and as a lipid feedstock [22], the present work aimed to evaluate its potential as a novel source for food and feed. Therefore, this microalga, produced at an industrial facility, was characterized in terms of its proximate and biochemical composition as well as antioxidant activity in vitro by means of different methodologies.

## 2. Results and Discussion

### 2.1. Proximate Composition

The macro composition of *Tetraselmis* sp. CTP4 is presented in Table 1. The biomass under study was compared with that of other *Tetraselmis* strains and to the well-known microalgae classified by EFSA as food ingredients, *Chlorella* and *Arthrospira*. The analysed biomass had low moisture content (~4 g/100 g) as compared to other microalgal biomasses (Table 1). Protein content was identical to the values previously reported for industrially grown *T. chui* (~31 g/100 g; [25]). Nevertheless, marine microalgae often present protein contents lower than those obtained in freshwater species (e.g., *Arthrospira* sp. and *Chlorella* sp.; Table 1), which can easily reach 50 to 65 g/100 g [26]. As previously reported for *Tetraselmis* sp. CTP4 (<10 g/100 g; [22]), lipid contents of 7 g/100 g were obtained upon cultivation under nutrient repletion (Table 1). Similar lipid contents were also observed in other strains of this genus [27] as well as in other microalgal strains (Table 1). Digestible carbohydrates accounted for 18 g/100 g of *Tetraselmis* sp. CTP4 biomass. Microalgae belonging to the genus *Tetraselmis* are known to accumulate significant amounts of carbohydrates, and, according to the results obtained, the strain under study might be a promising feedstock for the exploitation of biotechnological applications for this purpose. The content of dietary fibres, 25 g/100 g of biomass (Table 1), was considerably higher as compared to that of other *Tetraselmis* strains (2–3 g/100 g; [28]), and of *Chlorella* and *Arthrospira* (2–3 g/100 g; Table 1). The ash content of *Tetraselmis* sp. CTP4 was 15 g/100 g, being similar to the values obtained for other marine strains (Table 1) [25]. On the other hand, freshwater strains usually display lower ash contents, as its content in the final biomass varies according to the concentration of salt used for growth. Finally, the energetic/calorific value of the biomass produced is similar to that of *Arthrospira* (1241 kJ/100 g, 297 kcal/100 g). From a nutritional point of view, the energy value is low, mainly due to the low lipid content and the presence of significant amounts of fibres and ash.

**Figure 1:**
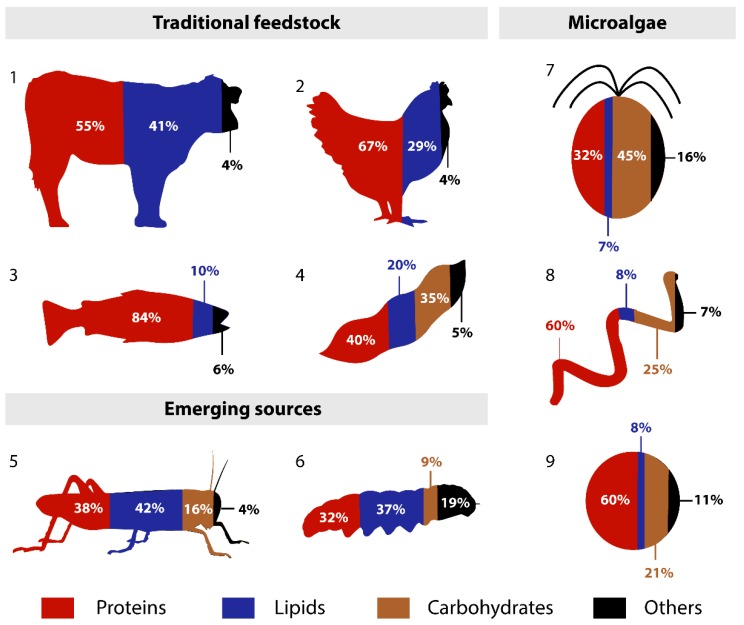
Illustrative comparison of the macronutritional composition (% of dry matter) for human and animal nutrition of traditional (1–4), emerging (5,6), and microalgal feedstocks (7–9). 1: Beef (FAO); 2: Chicken (USDA); 3: Fish (Bass; FAO); 4: Soybean (Seeds raw; USDA); 5: Grasshopper—*Ruspolia nitidula* [30]; 6: Black soldier fly prepupae—*Hermetia illucens* [31]; 7: *Tetraselmis* sp. CTP4 (Present work); 8: *Spirulina* (Dried; USDA)[29]; 9: *Chlorella vulgaris* (Allma product sheet)

In order to provide a better overview of the nutritional potential of *Tetraselmis* sp. CTP4, a comparison of the macronutritional composition with traditional, emerging, and microalgal feedstocks is shown in Figure 1. Overall, *Tetraselmis* sp. CTP4 biomass displays a proximate composition similar to that of soybean and other microalgae feedstocks, with higher carbohydrate and lower lipid contents than those commonly present in the traditional and emerging feedstocks presented in the figure. On the other hand, the protein content of *Tetraselmis* sp. CTP4 biomass (31 g/100 g) is lower than those of other microalgae and traditional animal feedstocks but closer to soybean and emerging sources (32–40 g/100 g). Regarding carbohydrates, it is noteworthy that the dietary fibres were a major component detected in the biomass of *Tetraselmis* sp. CTP4, which are important from a nutritional point of view, and are normally present in low amounts in the other feedstocks shown in Figure 1. The only exception is the amount of dietary fibres reported for *Ruspolia nitidula* (grasshopper) that range from 11.0% to 14.5% of the biomass dry weight [30]. Finally, the mineral fraction (ashes) was higher in *Tetraselmis* sp. CTP4 biomass than those commonly found in the other feedstocks discussed, with the exception of black soldier fly prepupae (*Hermetia illucens*), which contains a high mineral content of 19 g/100 g [31].

### 2.2. Amino Acid Profile

The amino acid (AA) profile is essential to assess the nutritional quality of a given food or feed. Regarding indispensable AA (IAA), according to the World Health Organization (WHO), the biomass of *Tetraselmis* sp. CTP4 shows high contents of leucine, valine, lysine, and phenylalanine (Table 2). *Tetraselmis* sp. CTP4 presented a lower amount of IAA when compared to other marine microalgae, such as *T. chui* and *Phaeodactylum tricornutum* [32], and some freshwater strains [29,32]. This difference might be explained by the lower amount of total AA found in strain CTP4 (10.7 g/100 g) when compared to *T. chui* (38.9 g/100 g), *Chlorella* sp. (35.6 g/100 g) and *Arthrospira* sp. (24.3 g/100 g; [29,32]). Analysing the AA profile (% of total AA), CTP4 shows high IAA levels (41% of total AA; Table 2), similar to those reported for *T. chui* (36.9%), *Chlorella* sp. (45.4%), and *Arthrospira* sp. (41.7%). Although the relative percentages of IAA are similar, some differences could be observed. Overall, higher relative abundances of leucine and valine were reported for *Chlorella* sp., when compared to the strains shown in Table 2. Industrially produced *Tetraselmis* sp. CTP4 displayed higher percentages of leucine, isoleucine, valine, lysine, threonine, and phenylalanine compared to *T. chui*, whereas tryptophan, histidine, and cysteine were detected at lower percentages [32]. The IAA profile of *Tetraselmis* sp. CTP4 is quite similar to that of *Arthrospira* sp., displaying higher levels of lysine but lower isoleucine, valine, and cysteine contents. Histidine was almost absent from the AA profile of *Tetraselmis* sp. CTP4, representing about 2% of the total AA of the remaining strains presented in Table 2. 

### 2.3. Lipid Profile

The fatty acid (FA) profile of *Tetraselmis* sp. CTP4 is mainly composed of palmitic (C16:0), palmitoleic (C16:1), oleic (C18:1), linoleic (C18:2), and α-linolenic (C18:3*n*-3) acids, which together are responsible for more than 80% of total FA (Table S1 [33,34]). Stearic (C18:0), hexadecatrienoic (C16:3*n*-3), and eicosapentaenoic (EPA; C20:5*n*-3) acids correspond to most of the remaining FA detected in *Tetraselmis* sp. CTP4. Polyunsaturated (PUFA) and monounsaturated (MUFA) fatty acids are the most abundant, while saturated fatty acids (SFAs) are present in lower amounts. The sum of PUFAs was equal to 36% of total FAs, whereas *n*-3 PUFA corresponded to about 17% of the total FAs, mainly represented by hexadecatrienoic (2.7% of total FAs), EPA (2.8% of total FAs), and α-linolenic acids (11.6% of total FAs), which are important for different nutritional applications. When compared to other microalgal strains, for example *Chlorella,* a higher amount of *n*-3 PUFAs can be found (Table S1), due to the high concentration of hexadecatrienoic and α-linolenic acids (12.7% and 32.9% of total FAs, respectively). However, the fatty acid profile of *Chlorella* lacks the long-chain *n*-3 PUFA (>20 carbons; e.g., EPA), which are generally absent from freshwater microalgal strains. In fact, EPA is a long-chain *n*-3 PUFA produced from marine biomass, being essential to several metabolic pathways in humans and animals and for an adequate nutrition of children, infants in particular. Overall, the FA profile reported here is similar to those reported for this strain in previous works [22,24], as well as to other strains belonging to the *Tetraselmis* genus (Table S1). Notable exceptions are the higher amounts of PUFAs, including those of *n*-3 PUFA, and the absence of stearidonic acid (C18:4*n*-3), when *Tetraselmis* CTP4 is compared with other *Tetraselmis* strains (Table S1).

### 2.4. Carbohydrates Composition

Sugar analysis showed that *Tetraselmis* sp. CTP4 biomass is composed mainly of glucose (Glc, 13.7 g/100 g), followed by galactose (Gal, 4.98 g/100 g) and mannose (Man, 1.33 g/100 g) in lower proportions (Table 3). Arabinose (Ara) and xylose (Xyl) were present in residual amounts (<0.2 g/100 g). This is in accordance with the literature, since glucose was the principal neutral sugar (75%–85%, Table 3), while lower levels of galactose (11%–16%), ribose (2%–5%), mannose (2%–3%), rhamnose, and arabinose (<1%) were detected in *T. chui* and *T. suecica* [35,36]. The main intracellular polysaccharide described in this genus is starch [36]. Starch is a storage polysaccharide common in green plants and algae. The two-keto-sugar acids have been described as the main sugars of *Tetraselmis* species, such as *T. striata* and *T. tetrathele*, due to the presence of the theca, an extracellular cell wall organized in multi-layered, fused scales [37]. The acid sugars were not determined. Compared to the genus *Tetraselmis*, higher amounts of xylose, mannose, and rhamnose were reported in *Arthrospira* sp. at the expenses of glucose and galactose [38].

Glycosidic-substitution analysis was performed to obtain more information about the structural characteristics of *Tetraselmis* polysaccharides (Table S2). The main linkages observed were 1,4-linked Glc (57 mol%) and 1,4-linked Gal (22 mol%). 1,4-Glc is substituted at C6 (1,4,6–Glc) with a content of 4.4 mol%, which confirms the presence of starch-like polysaccharides containing a high percentage of branching residues. The *Tetraselmis* polysaccharides seem to also be constituted by a galactan, with 1,4–Gal linkage in the backbone and substituted at C3, as inferred by the presence of 1,3,4–Gal (2 mol%). From a nutritional point of view, *Tetraselmis* sp. CTP4 is an interesting food as it could be a good source of energy provided by the starch-like polysaccharides.

### 2.5. Pigment Profile

Spray-dried *Tetraselmis* sp. CTP4 biomass (Table 4) contained high contents of chlorophyll (3531 mg/100 g), followed by neoxanthin (236 mg/100 g), lutein (226 mg/100 g), and violaxanthin (131 mg/100 g). Smaller quantities of zeaxanthin (11 mg /100 g) and *β*-carotene (8.4 mg/100 g) were also detected. Pigments can be added to foods as natural colouring agents and as antioxidants in healthy foods, to extend the shelf life and prevent oxidation during food processing. All photosynthetic microalgae contain chlorophyll, which usually ranges between 500 and 1500 mg/100 g of dry weight [39]. Interestingly, the chlorophyll contents of CTP4 dry biomass clearly exceeded this range (>3500 mg/100 g; Table 4). This high chlorophyll content may be beneficial to human health, since recent epidemiological studies provide evidence linking chlorophyll consumption to a decreased risk of colorectal cancer [40]. Although the most common industrial source of lutein is usually the marigold flower, the microalgae *Muriellopsis* spp., *Scenedesmus* spp., *Chlorella* spp., and *Chlorella protothecoides* present significant contents. *Tetraselmis* sp. CTP4 could also be a lutein source considering that its biomass contained about 0.2 g/100 g of this carotenoid (Table 4) and that improvement of the lutein contents of CTP4 might be achieved by the approach described in Cordero et al. [41] for *Chlorella sorokiniana* and an optimization of the growth conditions as suggested by recent trials, namely temperature and light intensity (manuscript submitted elsewhere). Carotenoid content optimization in *Tetraselmis suecica* has also recently been described upon the use of signalling molecules, such as salicylic acid, where contents of 4 mg/g DW were described [42].

Indeed, novel sources for this pigment might be important, because the lutein market size (USD 135 million in 2015) is estimated to generate significant gains in the near future [43]. A strong application outlook in eye health supplements may favour product demand, since lutein from microalgae (E161g) has been approved both in the EU and USA as a colour additive. The rising application of pigments in feed applications also accounted for over 30% of the carotenoid global demand in 2015, driven by growing consumer demand for meat, eggs, and salmon with a healthy appearance and standardized colouring. The natural carotenoids’ market size may see over 4% gains by 2024. Germany, France, the UK, and the USA are key contributing countries, favouring the expansion of the bioingredient industry. In this sense, the microalga *Tetraselmis* sp. CTP4 could be part of this demand for natural pigments, especially due to its content in chlorophyll and lutein.

### 2.6. Vitamin Profile

Ascorbic acid was the most abundant vitamin in *Tetraselmis* sp. CTP4 (79.2 mg/100 g), followed by tocopherol (20.28 mg/100 g) and niacin (7.98 mg/100 mg; Table 5). The vitamin C content of *Tetraselmis* sp. CTP4 biomass was higher than that reported for *Tetraselmis suecica* (19.1 mg/100 g; [45]) but lower than that of *Tetraselmis* sp. CS-362 (300 mg/100 g; [46]). The biomass of *Tetraselmis* sp. CTP4 also had intermediate levels of vitamin E (20.3 mg/100 g). However, in this case, the highest values were reported for *T. suecica* (20–50 mg/100 g; [47]) as compared to those of *Tetraselmis* sp. CS-362 (7 mg/100 g; [46]). Although no results have been reported for the contents of niacin in *Tetraselmis*, *Tetraselmis* sp. CTP4 showed a concentration slightly lower than usually found in microalgae (11–47 mg/100 g; [48]). Concerning the contents of the remaining vitamins, the values obtained here were lower than those described for *Tetraselmis* sp. and microalgae in general [45,46,47,48]. These low values may be a consequence of the fact that *Tetraselmis* sp. CTP4 biomass was processed under industrial conditions by means of spray-drying rather than freeze-drying. Heat inactivation of vitamins is a known process that depends on the matrix, pH, oxygen, light, and moisture [49]. As temperatures higher than 50 °C can be attained in the process of spray-drying, it is possible that some thermal decay took place.

### 2.7. Mineral Composition

Industrially produced biomass was mainly composed of the following minerals: Potassium (4.2%), magnesium (2.08%), calcium (1.19%), sodium (1.18%), and phosphorus (0.71%, Table S3 [26,29,32]). *Tetraselmis* sp. CTP4 presented higher magnesium and potassium contents when compared to the values previously reported for *T. chui*, *C. vulgaris*, and *Arthrospira* sp. Nevertheless, the phosphorus content observed in *Tetraselmis* sp. CTP4 was lower compared to those reported for *C. vulgaris* and *T. chui* [26,32]. Although there is a narrow threshold between the recommended and toxic levels of trace elements, the values observed for *Tetraselmis* sp. CTP4 are within the values commonly reported for other microalgal strains. Iron, copper, and zinc were detected at low concentrations (1.1–32.3 mg/100 g). Iron was the most abundant trace mineral in *Tetraselmis* sp. CTP4 (32.3 mg/100 g), with a concentration similar to that reported for *Arthrospira* sp. (28.5 mg/100 g). *T. chui* had a considerably higher concentration (173.4 mg/100 g), and *C. vulgaris* was reported to present considerably lower concentrations of this trace mineral (0.3 mg/100 g; [26]). Concentrations of zinc observed in CTP4 (2.9 mg/100 g) were similar to those of *C. vulgaris* (1.2 mg/100 g) and *Arthrospira* sp. (2.0 mg/100 g) but were lower than the values reported for *T. chui* (6.4 mg/100 g; Table S3).

Although low amounts of selenium and iodine were detected, it should be noted that both elements were not included in the industrial culture medium used for growth. Therefore, the addition of inorganic sources of both elements in the culture medium used for industrial production might allow improvement of the concentrations obtained in the final biomass product, as previously described for other chlorophytes [50]. Bioaccumulation of selenium has also been observed in *Tetraselmis* sp. CTP4, mainly in the form of selenomethionine (data not shown).

### 2.8. Antioxidant Activity

Upon extraction with solvents of different polarities, several in vitro assays were performed to determine the antioxidant activity of extracts obtained from the biomass produced in industrial photobioreactors. Values of antioxidant activity are presented as the half maximal inhibitory concentration (IC_50_) in mg/mL (Table 6). Ethyl acetate and acetone extracts showed higher radical scavenging activity (RSA) than those obtained with other solvents. The former extracts were more efficient in scavenging the 1,1-diphenyl-2-picrylhydrazyl (DPPH) radical (IC_50_ = 2.6 mg/mL) than the 2,2’-azino-bis(3-ethylbenzthiazoline-6-sulfonicacid) (ABTS) radical (IC_50_ = 6.9 mg/mL). Conversely, hexane, aqueous, and ethanolic extracts were not able to scavenge more than 50% of the free radicals when tested at 10 mg/mL. The same trend was observed for the ability of the extracts to reduce ferric iron (FRAP): Acetone (IC_50_ = 0.3 mg/mL) and ethyl acetate (IC_50_ = 0.5 mg/mL) extracts displayed the highest activities as compared to aqueous, ethanolic, and hexane extracts, whose IC_50_ were considerably higher (>1.1 mg/mL). The antioxidant contents of the samples tested might be related with the presence of phenolic compounds and/or carotenoid pigments. These compounds occur naturally in microalgae and many studies have demonstrated positive correlations between antioxidant activity and the concentration of these compounds [19]. Nevertheless, considering that microalgal extracts, particularly those using acetone and ethyl acetate, are generally more enriched in carotenoids than in phenolic compounds [6,17,19], the observed antioxidant activity is probably related with the carotenoids present in the extracts. Compounds with RSA have been in high demand, particularly those from natural sources, as replacements of synthetic antioxidant food preservatives, such as butylated hydroxytoluene (BHT, E321). This is mainly due to their protective role against oxidative stress and associated chronic disorders [51], and the safety concerns regarding the use of BHT in food and feed [52].

Chelation of redox metals, such as Fe and Cu, is also an effective way to prevent oxidative damage [53]. Hence, the same extracts were tested for their copper (CCA) and iron (ICA) chelating activities and compared to the known chelating agent, ethylenediamine tetraacetic acid (EDTA). All extracts were ineffective in chelating both copper and iron. The only exception was the acetone extract, which was able to chelate 50% of the initial iron concentration at 6.1 mg/mL. Oxidative stress can have implications in the rise and development of neurological disorders, such as Alzheimer’s disease; therefore, the chelation of redox metals for this ailment was previously proposed [53]. The results obtained with *Tetraselmis* sp. CTP4 were similar to those obtained by Custódio et al. [6], also with acetone extracts of another *Tetraselmis* strain, which displayed a similar ICA. On the other hand, our extracts did not show CCA, which is consistent with data previously reported for microalgae of the same genus [6,17]. It is possible that compounds with CCA are not present in this strain or that the production system (including biomass processing) may hamper the preservation of this bioactivity in the biomass. Nonetheless, *Tetraselmis* sp. CTP4 can still be a potential source of bioactive compounds with antioxidant activity.

### 2.9. Microbiological Evaluation

A detailed microbiological profile of the biomass produced in closed photobioreactors was achieved according to the European Legislation for Food (Table 7). Aerobic plate total counts and yeasts were 3.6 × 10^2^ and 1.0 × 10^2^ CFU/g, respectively. Enterobacteria and moulds were below the detection limits (<1 × 10^1^ CFU/g). The screened pathogenic bacteria were either below the detection limits or negative at 25 g. Overall, concerning microbiological specifications, *Tetraselmis* sp. CTP4 biomass was considered premium and free from pathogens although no microbiological criteria for microalgae is available in the EC NO 2073/2005.

### 2.10. Toxicological Evaluation

In order to fully understand the potential for nutritional purposes, both human and animal, a thorough toxicological evaluation was carried out in accordance with the most important contaminants proposed by the World Health Organization (Table 8). Therefore, several toxic metals were analysed, and the results obtained revealed that all were below the quantification limit, except for cadmium, which was present in only trace amounts (0.2 μg/g) in the analysed biomass (Table 8). Nevertheless, the cadmium content is below the limit regarded in the European legislation for foodstuffs (<3 mg/Kg; EU NO 488/2014). The cadmium detected in the biomass probably comes from the culture medium used in the industrial production, as the elemental analysis of the concentrated culture medium also revealed the presence of low cadmium levels (data not shown).

One important toxicological factor in industrially produced microalgal biomass is the presence of cyanotoxins. Cyanobacteria are common contaminants observed in large-scale production facilities, both in fresh- and salt-water systems. In accordance with the microscopic observations during the biomass production period where cyanobacteria were not detected [23], a screening for microcystins-LR, -RR, -LA, and cylindrospermopsin also revealed that they were absent from the produced biomass. We also evaluated the presence of mycotoxins that are common in some cereal grains. Therefore, aflatoxins B1, B2, G1, and G2 were analysed and the obtained results revealed that all were below the detection limit of the method (<0.5 ng/g). Finally, three distinct methods were used to analyse the presence of polycyclic aromatic hydrocarbons (PAHs; 9 compounds), organochlorine pesticides (24 compounds), and pesticides residues (about 250 residues). As no pesticides are used in the industrial production of microalgal biomass, the presence of PAHs and pesticides could only be due to their accumulation in the massive amounts of ground water used to produce the microalgal biomass. However, none of the analyses performed revealed any PAHs and pesticides in the industrially produced biomass.

Taken together, it can be concluded that industrially produced biomass is free from all common toxic factors tested, except for a residual amount of cadmium that can be eliminated from future production batches by using a different culture medium.

## 3. Materials and Methods

### 3.1. Microalgae Growth

*Tetraselmis* sp. CTP4 was previously isolated as described in Pereira et al. [22]. The growth in urban wastewater as well as in laboratory and industrial systems was published elsewhere [22,23,24]. Biomass was produced between 17 October and 14 November 2016, in 35- and 100-m^3^ industrial tubular photobioreactors, as described in Pereira et al. [23]. Briefly, *Tetraselmis* sp. CTP4 cultures were grown semi-continuously in both photobioreactors at a salinity of 20 g/L, using a culture velocity of 1 m/s and a pH set point for CO_2_ injection of 8.0. Produced biomass was concentrated in a Pall WUSP-6443 micro-filtration system and later dried in an MDR-150 high-speed centrifugal spray drier. In order to present an accurate quantification of the biochemical profile of *Tetraselmis* sp. CTP4, all results were normalized by removing the salt content of the biomass.

### 3.2. Proximate Composition

Total protein content was determined with a Foss Kjeltec 2200 protein analyser system, while total lipids were determined by Soxhlet extraction, followed by solvent evaporation in a Buchi R-210 rotary evaporator. Dietary fibres were determined according to the AOAC 991.43 and AOAC 985.29 norms. Ash content was determined by burning the samples at 540 °C for 6 h in a muffle furnace (Nabertherm B180 MB2). Digestible carbohydrates were calculated by difference, whereas energy was calculated using standard equations (Reg. EU Nº 1169/2011).

### 3.3. Amino Acid Profile

Amino acids were determined by ultra-performance liquid chromatography (UPLC) using the Waters Acquity UPLC equipped with an Accq-Tag Ultra C18 column (1.7-µm particle size (p.s.), 2.1 × 100 mm). The amino acids were released from protein by acid hydrolysis. The sulphur-containing amino acids, methionine, cystine, and cysteine, were first subjected to performic acid oxidation into methionine sulphone and cysteic acid. A separate hydrolysis with lithium hydroxide was performed to release tryptophan from the matrix.

### 3.4. Fatty Acid Profile

The profile of fatty acid methyl esters (FAMEs) was analysed according to the procedure described in Pereira et al. [16]. Briefly, samples were homogenized in a solution of methanol and acetyl chloride (20:1, *v/v*) with an IKA Ultra-Turrax T10B disperser for 2 min. Afterwards, samples were derivatised for 60 min at 90 °C and the FAMEs were sequentially extracted four times from the reaction mixture with *n*-hexane. The hexane extracts were dried with anhydrous sodium sulphate, filtered with a 0.2-µm filter (Whatman^®^ Puradisc, PTFE), and evaporated with a gentle stream of nitrogen. The dried residue was resuspended in 500 µL of hexane and stored at −20 °C until the gas chromatography (GC) analysis. FAMEs were analysed in a Bruker Scion 456/GC, Scion TQ MS coupled to a 30-m ZB-5MS capillary column with an internal diameter (i.d.) of 0.25 mm and film thickness of 0.25 μm (Phenomenex). Individual calibration curves were established for each FAME using Supelco^®^ 37 Component FAME Mix (Sigma-Aldrich, Sintra, Portugal).

### 3.5. Analysis of Carbohydrates

Neutral sugars were determined as alditol acetates by gas chromatography as described by Nunes et al. [54]. The monosaccharides were obtained after hydrolyses of the polysaccharides with sulphuric acid (1 M) at 100 °C for 2.5 hours. Monosaccharides were reduced with sodium borohydride and acetylated by acetic anhydride using methylimidazole as a catalyst. The alditol acetate derivatives formed were analysed by GC with a 30-m column DB-225 (i.d. of 0.25 mm and film thickness of 0.15 µm; J&W Scientific, Folsom, CA, USA) using a flame ionization detector (Perkin Elmer, Clarus 400). The monosaccharides were identified by the retention time and quantified using 2-deoxyglucose as an internal standard. The hydrolysis of all samples was done in duplicate and each one was injected at least twice.

Glycosidic-substitution analysis was determined by gas chromatography-quadrupole mass spectrometry (GC-qMS) of the partially methylated alditol acetates (PMAAs) as described in Oliveira et al. [55]. Samples were methylated with CH_3_I in alkaline medium. The methylated sample was hydrolysed with 2 M trifluoroacetic acid (1 mL) at 120 °C for 1 h, and then reduced and acetylated as previously described for neutral sugar analysis (using NaBD_4_ instead of NaBH_4_). The PMAAs were separated and analysed by GC–qMS (GC-2010 Plus, Shimadzu, Duisburg, Germany). The GC was equipped with a DB-1 (J&W Scientific, Folsom, CA, USA) capillary column (30-m length, 0.25-mm i.d., and 0.10-µm film thickness). The GC was connected to a GCMS-QP 2010 Ultra Shimadzu mass quadrupole selective detector operating with an electron impact mode at 70 eV and scanning the range m/z 50–700 in a 1-s cycle in a full scan mode acquisition.

### 3.6. Determination of Pigments

The pigments profile was determined according to Wright et al. [56] using a Waters Alliance 2695 HPLC and Waters 2996 photodiode array detector (PAD) coupled to a Waters Spherisorb column (5 µm, 4.6 × 250 mm). Briefly, samples were extracted with methanol, filtered through 0.2-µm syringe filters, and injected in the HPLC. The standards of alloxanthin, diatoxanthin, lutein, neoxanthin, violaxanthin, and zeaxanthin were obtained from DHI Lab Products (Hørsholm, Denmark), while *β*-carotene was supplied by Sigma-Aldrich.

### 3.7. Determination of Vitamins

All vitamins were determined with an Agilent Technologies 1200 Series HPLC UV/VIS unless stated in contrary. Vitamins A and E were determined using a Kromasil 100-5-SIL column (5 µm, 4.6 × 250 mm), according to the EN 12823-1:2000 and EN 12822:14 standards, respectively. Samples for the determination of vitamin A were saponified with an ethanolic solution of sodium hydroxide and the extraction was carried out with *n*-hexane. Vitamin C was determined using a Waters Spherisorb column (5 µm, 4.6 × 250 mm) according to the EN 14130 standard. The extraction was performed with a solution of meta-phosphoric acid followed by a reduction of L(+)-dehydroascorbic and L(+)-ascorbic acids with a solution of L-cysteine. Vitamins B1 (thiamine), B2 (riboflavin), and B6 were determined using a fluorescence detector (HPLC-FD) coupled to an Atlantis dC18 column (5 μm, 4.6 × 150 mm) according to the Waters technical note (Vitamins B1 and B2) and the EN 14164:2008 standard (Vitamin B6). All samples were treated with hydrochloric acid followed by enzymatic digestion with clara-diastasa (Sigma-Aldrich). A Licrospher 60 Rp-select B column (5 µm, 4.0 × 125 mm) was used to determine the content of vitamin B3 (niacin) in the samples, according to the EN 15652 standard.

Vitamin B5 (panthotenic acid) was determined by LC-MS-MS using Micromass Quattro Micro API y SCIEX Triple Quad 5500 coupled to a Zorbax Eclipse XDB-C8 column (3.5 μm, 3.0 × 100 mm). Vitamins B9 (folic acid) and B12 (cobalamin) were concentrated with immunoaffinity columns (Biopharm Rhône LTD) and an Atlantis dC18 column (5 μm, 4.6 × 150 mm) was used.

### 3.8. Mineral Analysis

The mineral composition was determined by the ALS Group, with a Varian 730-ES atomic emission spectrometry with inductively coupled plasma (ICP-OES) as per ISO 11885:2007. Iodine was determined by ICP-OES according to the EN 15111:2007 standard. Stoichiometric calculations of concentrations were established from measured values. All samples were prepared according to the CZ_SOP_D06_02_J02 (chap. 10.17.1, 10.17.2, 10.17.4, 10.17.7, 10.17.8). Prior to analysis, samples were homogenized and mineralized by acids and hydrogen peroxide.

### 3.9. Evaluation of In Vitro Biological Activities

For the evaluation of bioactivities, industrially produced biomass was extracted with selected solvents of different polarities, namely hexane, ethyl acetate, acetone, ethanol, and distilled water. Homogenization was achieved by means of a disperser IKA Ultra-Turrax T10B, while the extraction occurred overnight at room temperature. All extracts were filtered through 0.7-μm pore glass fibre filters (VWR) and further concentrated in a rotatory evaporator (IKA, RV10 digital, Staufen, Germany) at 40 °C under reduced pressure. Extracts were resuspended in DMSO and stored at −20 °C.

Extracts were evaluated for their antioxidant potential through complementary in vitro assays, namely radical scavenging activity on DPPH and ABTS radicals, ferric reducing antioxidant power (FRAP), and metal chelating activities (MCA) on Cu^2+^ and Fe^2+^, using the methods described in Custódio et al. [6] and Rodrigues et al. [57]. BHT and EDTA were used as positive controls for the radical scavenging activity (RSA) and FRAP, and MCA, respectively.

### 3.10. Microbiological Evaluation

All microbiological analyses were performed in laboratories certified by the ISO 17025. Briefly, microalgal biomass samples were serially diluted in Ringer’s solution and triplicates of the dilutions were plated on the appropriate media. Total counts of aerobic microorganisms were assessed in plate count agar incubated for three days at 30 °C (EN ISO 4833-1:2013). Enterobacteria were determined in violet red bile dextrose agar incubated at 37 °C for 24 hours (EN-ISO 8523:1991), while yeasts and moulds were spread-plated in rose-bengal chloramphenicol agar and incubated for five days (NP 3277-1:1987). *Escherichia coli* (ISO 16649-2:2001) and *Staphylococcus aureus* (ISO 6888-2:1999) were respectively analysed by plating in MacConkey agar and Baird-Parker agar supplemented with rabbit plasma fibrinogen and incubated at 37 °C for three days. The presence of *Listeria monocytogenes* was evaluated using tryptone soya yeast extract agar and sheep’s blood agar after incubation at 37 °C for 1 to 3 days (EN ISO 11290-1:1996). *Salmonella* spp. was assessed in brilliant green agar and xylose lysine desoxycholate agar upon incubation at 37 °C for 24 h (EN ISO 6579:2002). The presence of *Pseudomonas* spp. was performed using cetrimide fusidin cephaloridine agar and incubation at 30 °C for two days (ISO 13720:2010), while the occurrence of *Vibrio* spp. was carried out in thiosulfate citrate bile sucrose agar after an incubation at 37 °C for 24 h (ISO/TS 21872-2:2007).

### 3.11. Toxicological Evaluation

Determination of toxic metals was performed as described for the mineral analysis (Section 3.7). Cyanotoxins were analysed by the EPA Method 544 using a Liquid Phase Chromatograph Finnigan Surveyor (Thermo Scientific, San Jose, CA, USA), coupled with a spectrometry detector (MS Mass LCQ FleetTM ion trap), with an electrospray (ESI) interface and a C18 Hypersil Gold column (100 × 4.6 mm I.D., 5 μm, ThermoScientific, Waltham, MA, USA). The absence of microcystins-LR, -RR, -LA, and cylindrospermopsin was confirmed by the non-existence of the precursor ion for each cyanotoxin, 995.5[M + H]^+^, 519.9 [M + 2H]^2+^, 910.5 [M + H]^+^, and 416.5 [M + H]^+^, respectively. Aflatoxins B1, B2, G1, and G2 were determined using an Agilent Technologies 1200 series HPLC coupled to a SPHERISORB column (4.6 × 250 mm, 5 µm ODS2, Waters) according to ISO16050:2003. The analysis of PAHs and pesticides was performed by Silliker Portugal S.A., using certified methods. PAHs were analysed using a 7890 Agilent GC-MS equipped with a J&W VF-17 MS column (30 m × 0.25 mm, 0.25 μm, Agilent) according to F013550.0. Pesticides, both organochlorine (25 pesticides) and residues (about 250 pesticides), were evaluated using an Agilent 7890 gas chromatograph coupled to a 7000 Series MS according to the PS1052 e PS0001110 methods, respectively.

## 4. Conclusions

The biomass of *Tetraselmis* sp. CTP4 produced in an industrial facility displayed a composition comparable to that of other strains belonging to the genus *Tetraselmis*, as well as to other microalgal genera (e.g., *Chlorella* and *Arthrospira*), generally regarded as safe to be consumed as food, because of their track record spanning at least half a century. The same can be said when *Tetraselmis* sp. CTP4 was compared to food crops (e.g., soybean) and other emerging feedstocks. Its biomass displayed interesting contents in terms of protein, dietary fibres, carotenoids, and vitamins coupled with a moderate antioxidant capacity. In addition, the microbiological and toxicological evaluation revealed that most common pathogens and toxic compounds were absent from the industrially produced biomass. As *Tetraselmis* sp. CTP4 is able to grow in industrial photobioreactors at temperatures as high as 35 °C and salinities close to that of seawater, this microalga can be considered as a suitable feedstock for human and animal nutrition, particularly in countries where these two abiotic factors can be important hindrances for the production of traditional crops. The search for alternatives to the latter is crucial when freshwater is becoming a scarce commodity, the average temperatures are on the rise, and non-animal feedstocks are increasingly needed to accommodate the demand by emergent markets, such as vegan food and feed.

## Figures and Tables

**Table 1:** Proximate composition of *Tetraselmis* sp. CTP4 grown semi-continuously in industrial tubular photobioreactors (g/100 g). Values represent the mean and corresponding standard deviation (*n* = 3). Values from the literature for *Tetraselmis chui*, *Tetraselmis suecica*, *Chlorella vulgaris* and *Arthrospira* sp. are also presented. n.r.—not reported.

Contents (g/100 g)	*Tetraselmis* sp. CTP4	*Tetraselmis chui* ^1^	*Tetraselmis suecica* ^2^	*Chlorella* *vulgaris* ^3^	*Arthrospira* sp. ^4^
Moisture	3.88 ± 0.35	<7	n.r.	4.9	4.7
Protein	31.20 ± 0.48	35–40	48.7	56.9	57.5
Lipids	7.04 ± 0.42	5–8	8.0	7.5	7.7
Digestible carbohydrates	18.08 ± 4.18	30–32	22.4	19.2	20.3
Dietary fibres	24.60 ± 3.85	2–3	3.4	0.5	3.6
Ash	15.20 ± 0.80	14–16	17.5	10.9	6.2
Energy (kJ/100 g)	1241 ± 49	n.r.	n.r.	n.r.	1213
Energy (kcal/100 g)	297 ± 12	n.r.	n.r.	n.r.	290

^1^ Fitoplancton Marino S.L. ^2^ Tulli et al. [28]. ^3^ Allma product sheet. ^4^ United States Department of Agriculture. [29]

**Table 2:** Amino acid concentration (g/100 g DW) of *Tetraselmis* sp. CTP4 grown semi-continuously in industrial tubular photobioreactors. Values represent the mean and corresponding standard deviation (*n* = 3). Values in brackets represent the % of total amino acid. Values from the literature for *Tetraselmis chui*, *Chlorella* sp. and *Arthrospira* sp. are also presented in % of total amino acid. n.r.—not reported.

Amino acid	*Tetraselmis* sp. CTP4	*Tetraselmis* *chui* ^1^	*Chlorella* sp. ^1^	*Arthrospira* sp. ^2^
*Indispensable amino acids (IAA)*				
Leucine	2.28 ± 0.02 (8.83)	7.5	9.9	8.5
Isoleucine	1.12 ± 0.02 (4.34)	3.5	4.8	5.5
Valine	1.55 ± 0.02 (6.01)	4.9	6.8	6.0
Lysine	1.70 ± 0.09 (6.59)	5.7	6.6	5.2
Threonine	1.27 ± 0.05 (4.92)	4.1	5.1	5.1
Tryptophan	0.37 ± 0.03 (1.43)	2.4	1.0	1.6
Methionine	0.61 ± 0.03 (2.36)	2.5	2.8	2.0
Phenylalanine	1.44 ± 0.08 (5.58)	4.8	6.0	4.8
Histidine	0.04 ± 0.01 (0.15)	1.6	2.3	1.9
Cystine + Cysteine	0.28 ± 0.01 (1.08)	2.9	n.r.	1.1
**Total IAA**	**10.7 (41.3)**	**39.8**	**45.4**	**41.7**
*Non-indispensable amino acids (NIAA)*				
Alanine	2.04 ± 0.06 (7.90)	6.1	9.2	7.1
Arginine	1.70 ± 0.04 (6.59)	9.6	7.0	10.0
Aspartic acid (Asx)	2.89 ± 0.02 (11.2)	14.4	9.9	14.4
Glutamic acid (Glx)	3.64 ± 0.02 (14.1)	12.3	12.4	5.3
Glycine	1.58 ± 0.06 (6.12)	6.7	6.6	4.1
Proline	1.26 ± 0.03 (4.88)	3.7	5.4	5.2
Serine	1.19 ± 0.08 (4.61)	4.3	4.2	4.4
Tyrosine	0.85 ± 0.01 (3.29)	3.1	n.r.	8.5

^1^ Calculated from Tibbetts et al. [32]. ^2^ United States Department of Agriculture [29].

**Table 3:** Sugar composition of *Tetraselmis* sp. CTP4 grown semi-continuously in industrial tubular photobioreactors (g/100 g). Values represent the mean and corresponding standard deviation (*n* = 3). Values in brackets represent the mol% of total sugars. Values from the literature for *Tetraselmis chui*, *Tetraselmis suecica*, and *Arthrospira* sp. are also presented in % of total sugars. v.—vestigial (<0.1 g/100g); n.d.—not detected; n.r.—not reported.

Contents	*Tetraselmis* sp. CTP4	*Tetraselmis chui* ^1^	*Tetraselmis suecica* ^1^	*Arthrospira* sp. ^2^
Arabinose	0.18 ± 0.01 (1.0)	0.41	0.90	n.r.
Xylose	0.10 ± 0.01 (0.6)	n.d.	n.d.	7.0
Mannose	1.33 ± 0.02 (6.5)	1.8	3.0	9.3
Galactose	4.98 ± 0.03 (24.5)	11.3	15.7	2.6
Glucose	13.68 ± 0.07 (67.3)	84.7	74.8	54.4
Rhamnose	v.	0.04	0.97	22.3
Ribose	v.	1.8	4.5	n.r.
Others	-	n.r.	n.r.	4.3

^1^ Brown [35]. ^2^ Shekharam et al. [38].

**Table 4:** Pigment profile of *Tetraselmis* sp. CTP4 grown semi-continuously in industrial tubular photobioreactors (mg/100 g). Values represent the mean and corresponding standard deviation (*n* = 3). Values from the literature for *Tetraselmis chui*, *Tetraselmis* sp. M8, and *Chlorella vulgaris* are also presented. n.d.—not detected; n.r.—not reported.

Pigments (mg/100 g)	*Tetraselmis* sp. CTP4	*Tetraselmis chui* ^1^	*Tetraselmis* sp. M8 ^1^	*Chlorella vulgaris* ^2^
Chlorophyll *a* and *b*	3531.2 ± 152.1	n.r.	n.r.	2600
Violaxanthin	130.8 ± 5.7	54.6	22.9	n.r.
Antheraxanthin	n.d.	20.1	12.6	n.r.
Neoxanthin	236.4 ± 11.9	n.d.	n.d.	n.r.
Zeaxanthin	10.8 ± 1.3	n.d.	n.d.	626
Lutein	225.6 ± 8.5	62.4	66.5	1011
*α*-carotene	n.d.	17.4	3.0	6.92
*β*-carotene	8.4 ± 0.7	94.1	105.7	8.26

^1^ Ahmed et al. [44]. ^2^ Allma product sheet.

**Table 5:** Vitamin contents of *Tetraselmis* sp. CTP4 biomass grown semi-continuously in industrial tubular photobioreactors. Values from the literature for *Tetraselmis* sp. as well as *Chlorella vulgaris* and *Arthrospira* sp. are also presented. n.d.— not detected; n.r.—not reported.

Vitamins	*Tetraselmis* sp. CTP4	*Tetraselmis* sp. CS-362 ^1^	*Chlorella vulgaris* ^2^	*Arthrospira* sp.^3^
A—Retinol (µg/100 g)	<4	220	<20	29
B1—Thiamin (mg/100 g)	0.18	10.9	0.03	2.38
B2—Riboflavin (mg/100 g)	0.53	2.6	0.05	3.67
B3—Niacin (mg/100 g)	7.98	n.r.	0.10	12.8
B5—Pantothenic Acid (mg/100 g)	0.65	n.r.	0.08	3.48
B6—Pyridoxal phosphate (mg/100 g)	6.9	0.6	0.08	0.36
B7—Biotin (mg/100 g)	n.d.	0.13	n.r.	n.r.
B9—Folic acid (µg/100 g)	0.02	2000	30.6	94
B12—Cianocobalamin (µg/100 g)	7.8	195	0.10	-
C—Ascorbic acid (mg/100 g)	79.2	300	<100	10.1
E—Tocopherol (mg/100 g)	20.28	7	6.57	5

^1^ Brown et al. [46]. ^2^ Allma product sheet. ^3^ United States Department of Agriculture [29].

**Table 6:** Radical scavenging activity on the DPPH and ABTS radicals, ferric reducing antioxidant power (FRAP), and metal-chelating activity on copper (CCA) and iron (ICA) of organic and water extracts of *Tetraselmis* sp. CTP4. Results are expressed as the mean IC_50_ (mg/mL) and corresponding standard deviation (*n* = 4).

Sample	DPPH	ABTS	FRAP	CCA	ICA
Hexane	>10	>10	1.1 ± 0.1	>10	>10
Ethyl acetate	2.6 ± 0.2	6.9 ± 0.4	0.5 ± 0.0	>10	>10
Acetone	4.9 ± 0.3	8.7 ± 0.3	0.3 ± 0.0	>10	6.1 ± 0.2
Ethanol	>10	>10	1.1 ± 0.1	>10	>10
Water	>10	>10	>10	>10	>10
BHT	0.14 ± 0.01	0.11 ± 0.01	-	-	-
EDTA	-	-	-	0.08 ± 0.01	0.03 ± 0.00

**Table 7:** Microbiological evaluation of *Tetraselmis* sp. CTP4 biomass grown semi-continuously in industrial tubular photobioreactors. CFU = colony-forming unit.

	*Tetraselmis* sp. CTP4
Aerobic plate total counts (30 °C)	3.6 × 10^2^ CFU/g
Enterobacteria	<1.0 × 10^1^ CFU/g
*Staphylococcus aureus*	<1.0 × 10^1^ CFU/g
*Listeria monocytogenes*	<1.0 × 10^1^ CFU/g
*Escherichia coli*	<1.0 × 10^1^ CFU/g
*Salmonella* spp.	Negative (25 g)
*Pseudomonas* spp.	<1.0 × 10^1^ CFU/g
*Vibrio* spp.	Negative (25 g)
Yeasts (25 °C)	1.0 × 10^2^ CFU/g
Moulds (25 °C)	<1.0 × 10^1^ CFU/g

**Table 8:** Toxicological evaluation of *Tetraselmis* sp. CTP4 biomass grown semi-continuously in industrial tubular photobioreactors.

Toxic Substances	*Tetraselmis* sp. CTP4
**Toxic metals**	
Lead	<0.10 µg/g
Cadmium	0.2 ± 0.0 µg/g
Mercury	<0.10 µg/g
Arsenic	<0.80 µg/g
Tin	<2.50 µg/g
**Cyanotoxins**	
Microcystin LR	n.d.
Microcystin RR	n.d.
Microcystin LA	n.d.
Cylindrospermopsin	n.d.
**Mycotoxins**	
Aflatoxins B1 and B2	<0.5 ng/g
Aflatoxins G1 and G2	<0.5 ng/g
**Dioxins**	
Benzo[a]pyrene	<0.5 ng/g
Benzo[a]anthracene	<0.5 ng/g
Benzo[b]fluoranthene	<0.5 ng/g
Chrysene	<0.5 ng/g
Other polycyclic aromatic hydrocarbons ^1^	<0.5 ng/g
**Pesticides**	
Organochlorine pesticides ^2^	<0.01 µg/g
Screening of >200 residues ^3^	<0.01 µg/g

^1^ Benzo(k)fluoranthene, Indeno[1,2,3-cd]pyrene, Dibenzo[a,h]anthracene, Benzo[ghi]perylene, Benzo[a]pyrene. ^2^ 2,4′-DDD, 2,4′-DDE, 2,4′-DDT, 4,4′-DDD, 4,4′-DDE, 4,4′-DDT, Sum of DDD, DDE, DDT, Aldrin, Dieldrin, Sum of Aldrin and Dieldrin, alpha-Endosulfan, beta-Endosulfan, Endosulfan sulfate, Endosulfan (Sum of alpha- and beta-isomers and Endosulfan-sulphate), Hexachlorocyclohexane (HCH) alpha-isomer, Hexachlorocyclohexane (HCH) beta-isomer, delta-Hexachlorocyclohexane (delta-HCH), Lindane (Gamma-isomer (HCH)), Hexachlorocyclohexane (HCH), sum of isomers, except the gamma isomer, cis-Chlordane, Trans-Chlordane, Chlordane (sum of cis- and trans-Chlordane), cis-Heptachlor epoxide, trans-Heptachlor, epoxide, Heptachlor, Heptachlor (Sum of Heptachlor and Heptachlor epoxide), Endrin, Hexachlorobenzene (HCB), Isodrin, Metoxychlor. ^3^ Metalaxyl and metalaxyl-M (metalaxyl including other mixtures of constituent isomers including metalaxyl-M (sum of isomers)), Ethofumesate-2-keto, Ethofumesate, 3-Hydroxycarbofuran, Carbofuran, Sum of Carbofuran (including any carbofuran generated from carbosulfan, benfuracarb or furathiocarb) and 3-OH carbofuran), Abamectin, Acephate, Acetamiprid, Gibberellic acid, Aldicarb, Aldoxycarb, Aldicarb sulfoxide, Sum of Aldicarb, Haloxyfop-r-methyl, Aminocarb, Amitraz, n-(2,4-Dimethylphenyl)formamide, n-2,4-Dimethylphenyl-n´-methylformadine, n-2,4-Dimethylphenyl-n´-methylformanidine, Amitraz (amitraz including the metabolites containing the 2,4 -dimethylaniline moiety), Ancymidol, Asulam, Atrazine, Azadirachtin, Azinphos-ethyl, Azinphos-methyl, Azoxystrobin, Benalaxyl, Bendiocarb, Benfuracarb, Resmethrin, Boscalid, Bupirimate, Bupofrezin, Butocarboxim, Butralin, Cadusafos, Carbaryl, Carbendazim + Benomyl, Thiophanate-methyl, Sum of MBC, Carboxin, Carbosulfan, Cyanazine, Cyazofamid, Cycloate, Cymiazole hydrochloride, Cymoxanil, Cinidon ethyl, Cyproconazole, Cyprodinil, Cyromazine, Clofentezine, Clomazone, Cloquintocet-1-methylhexyl ester, Chlorantranquiliprole, Chlorfluazuron, Chloridazon, Chlortoluron, Clothianidin, Thiamethoxan, Sum of thiamethoxan and clothianidin, Dementon-s-methyl, Dementon-s-methyl sulfone, Dementon-s-methyl sulfoxide, Sum of dementon-s-methyl + demeton-s-methyl sulfoxide, Desethylatrazine, Terbuthylazine-desethyl, Desmedipham, Desmethyl pirimicarb, Desmethylformamido pirimicarb, Pirimicarb, Sum of pirimicarb, Diafenthiuron urea, Diallate, Diazinon, Diclofuanide, Diclofop methyl, Dicrotophos, Diethofencarb, Diphenamid, Diflubenzuron, Diflufenican, Dimethenamid-p (dimethenamid-p including other mixtures of constituent isomers (sum of isomers)), Dimethenamide, Dimethoate, Omethoate, Sum of dimethoate and omethoate, Dimethomorph, Dinotefuran, Disulfoton, Dissolfoton sulfone, Disulfoton sulfoxide, Sum of Disulfoton, Diuron, Dodine, Emamectin benzoate B1a, Heptenophos, Hexaconazole, Ethiofencarb, Ethiofencarb sulfone, Ethion, Ethofenprox, Hexythiazox, Famoxadone, Fenamidone, Fenamiphos, Fenamiphos sulfone, Fenamiphos sulfoxide, Sum of fenamiphos, Fenazquin, Fenbuconazole, Fenhexamid, Phenmedipham, Fenoxaprop-p-ethyl, Fenoxycarb, Fenpyroximate, Fenpropathrin, Fenpropridin, Fenpropimorph, Fenthion, Fenthion sulfone, Fenthion sulfoxide, Fenthion oxon, Fenthion oxon sulfone, Fenthion oxon sulfoxide, Sum of fenthion, Fenuron, Fipronil, Flonicamid, Florasulam, Fluazifop-p-butyl, Fluazifop-p, Flufenacet, Flufenoxuron, Fluquinconazole, Flurprimidol, Flutriafol, Fonofos, Forchlorfenuron, Formetanate, Formothion, Phosphamidon, Phosmet, Fosthiazate, Furalaxyl, Furathiocarb, Imazalil, Imazamethabenz -methyl, Imazamox, Imazethapyr, Imidacloprid, Indoxacarb, Iprovalicarb, Isopropalin, Isoproturon, Kresoxim-methyl, Lenacil, Linuron, Malaoxon, Malathion, Sum of malathion and malaoxon, Mandipropamid, Mepanipyrim, Methabenzthiazuron, Methamidophos, Metamitron, Metazachlor, Methidathion, Methiocarb, Methiocarb sulfone, Methiocarb sulfoxide, Sum of methiocarb, Metobromuron, Methomyl, Thiodicarb, Sum of methomyl and thiodicarb, Methoxyfenozide, Metoxuron, Metribuzin, Mevinphos, Myclobutanil, Milbemectin A3, Milbemectin A4, Monocrotophos, Monolinuron, Monuron, Neburon, Oxadiazon, Oxamyl, Oxycarboxin, Paclobutrazol, Paraoxon, Pencycuron, Pendimethalin, Picolinafen, Pymetrozine, Piperonyl butoxide, Pyraclostrobin, Pyrethrins, Pyridaben, Pyridalyl, Pyridate, Pyrimethanil, Pirimiphos-ethyl, Pirimiphos-methyl, Pyriproxyfen, Prochloraz, Profenofos, Promecarb, Propachlor, Propamocarb, Propanil, Propaquizafop, Propiconazole, Propyzamide, Propoxur, Quinalphos, Quinoxyfen, Quizalofop-ethyl, Rotenone, Simazine, Spinosad, sum of spinosyn A and spinosyn D, Spirotetramat, Spiroxamine, tau-Fluvalinate, Tebuconazole, Tebufenozide, Tebufenpyrad, TEPP, Terbufos, Terbuthylazine, Tetraconazole, Thiabendazole, Thiacloprid, Thiobencarb, Tiocarbazil, Thiram, Tolyfluanid, Triadimefon, Triadimenol, Sum of tradimefon + triadimenol, Triazamate, Tricyclazole, Trichlorfon, Tridemorph, Trifloxystrobin, Triflumizole, Trioforine, Vamidothion, Zoxamide.

## Supplementary Materials

The following are available online at https://www.mdpi.com/1420-3049/24/17/3192/s1, Table S1: Fatty acid profile of *Tetraselmis* sp. CTP4 grown in an industrial production facility., Table S2: Glycosidic-linkage analysis (mol%) of *Tetraselmis* sp. CTP4 grown semi-continuously in industrial tubular photobioreactors., Table S3: Composition of minerals and heavy metals of *Tetraselmis* sp. CTP4 biomass grown semi-continuously in industrial tubular photobioreactors.
