# Supplementary material for: Nutritional Potential and Toxicological Evaluation of Tetraselmis sp. CTP4 Microalgal Biomass Produced in Industrial Photobioreactors
Source: Molecules. 2019 Sep 3;24(17):3192. doi: 10.3390/molecules24173192 (PMC6749414; doi:10.3390/molecules24173192)
Supplement: Supplementary file 1 [file molecules-24-03192-s001.pdf]

SUPPLEMENTARY DATA for

Nutritional Potential and Toxicological Evaluation of *Tetraselmis* sp. CTP4 Microalgal Biomass Produced in Industrial Photobioreactors

Hugo Pereira [1], Joana Silva [2], Tamára Santos [1], Katkam N. Gangadhar [1,3], Ana Raposo [1], Cláudia Nunes [4,5], Manuel A. Coimbra [4,5], Luísa Gouveia [6], Luísa Barreira [1] and João Varela [1,*]

[1] CCMAR - Centre of Marine Sciences, University of Algarve, Gambelas, 8005-139 Faro, Portugal
[2] CMP - Cimentos Maceira e Pataias, ALGAFARM - Unidade de Produção de Microalgas, 2445-411 Pataias, Portugal
[3] LEPABE - Department of Chemical Engineering, Faculty of Engineering, University of Porto, 4200-465 Porto, Portugal
[4] CICECO – Aveiro Institute of Materials & [5] QOPNA, Department of Chemistry, University of Aveiro, 3810-193 Aveiro, Portugal
[6] LNEG - Laboratório Nacional de Energia e Geologia, I.P./Bioenergy Unit, Estrada do Paço do Lumiar 22, 1649-038 Lisbon, Portugal
* Correspondence: jvarela@ualg.pt; Tel.: +351 289 800 900; Fax: +351 289 800 069

Table S1 - Fatty acid profile of *Tetraselmis* sp. CTP4 grown in an industrial production facility. Values from the literature for *Tetraselmis chui, Tetraselmis suecica* and *Chlorella* sp. are also presented. Values represent the mean % of total fatty acids and corresponding standard deviation (n = 3).

**FAME**	***Tetraselmis* sp. CTP4**	***Tetraselmis chui*[1]**	***Tetraselmis suecica*[2]**	***Chlorella* sp.[1]**
C14:0	0.91 ± 0.01	0.2	1.3	0.6
C15:0	n.d.	n.d.	n.d.	0.5
C16:0	20.72 ± 1.18	19.9	16.0	21.8
C17:0	1.68 ± 0.03	n.d.	n.d.	n.d.
C18:0	2.09 ± 0.02	0.3	3.1	0.8
C21:0	1.74 ± 0.21	n.d.	n.d.	n.d.
C24:0	n.d.	0.1	n.d.	0.3
**∑ SFA**	**27.14**	**20.5**	**20.4**	**24.1**
C16:1	10.33 ± 0.92	3.6	3.3	7.9
C18:1	25.37 ± 1.56	9.8	27.2	3.6
C20:1	0.90 ± 0.01	2.5	2.3	n.d.
**∑ MUFA**	**36.60**	**15.9**	**32.8**	**11.5**
C16:2 n-6	1.13 ± 0.02	0.1	n.d.	4.4
C16:3 n-3	2.72 ± 0.10	1.5	n.d.	12.7
C16:4 n-3	n.d.	15.9	n.d.	n.d.
C18:2 n-6	16.51 ± 1.03	4.6	6.4	14.1
C18:3 n-3	11.60 ± 0.95	25.2	16.3	32.9
C18:3 n-6	0.50 ± 0.01	0.4	n.d.	n.d.
C18:4 n-3	n.d.	6.1	10.4	n.d.
C20:3 n-3	n.d.	0.1	n.d.	n.d.
C20:4 n-6	0.98 ± 0.02	1.2	1.5	n.d.
C20:5 n-3	2.82 ± 0.03	8.0	12.2	n.d.
**∑ PUFA**	**36.26**	**63.1**	**46.8**	**64.1**
**∑ n-3**	**17.14**	**56.8**	**40.5**	**45.6**

[1] *Dunstan et al.* [33]

[2] *Patil et al.* [34]

n.d. – not detected

Table S2 - Glycosidic-linkage analysis (mol%) of *Tetraselmis* sp. CTP4 grown semi-continuously in industrial tubular photobioreactors.

**Sugar residues**	**Mol%**
t-Xyl	1.1
Total Xyl	***1.1***
1,2,3,5-Araf	3.5
Total Ara	***3.5***
1,3,6-Man	2.8
Total Man	***2.8***
1,4-Gal	21.5
1,3,4-Gal	1.8
Total Gal	***26.0***
t-Glc	6.7
1,4-Glc	57.4
1,6-Glc	0.7
1,4,6-Glc	4.4
Total Glc	***69.2***

Table S3 - Composition of minerals and heavy metals of *Tetraselmis* sp. CTP4 biomass grown semi-continuously in industrial tubular photobioreactors. Values represent the mean and corresponding standard deviation (*n*=3). Values from the literature for *Tetraselmis chui, Chlorella vulgaris* and *Arthrospira* sp. are also presented.

	***Tetraselmis* CTP4**	***Tetraselmis chui*[1]**	***Chlorella vulgaris*[2]**	***Arthrospira sp.*[3]**
**Minerals (g/100 g)**				
Calcium	1.19 ± 0.17	2.99	0.59	0.12
Magnesium	2.08 ± 0.30	0.43	0.34	0.20
Phosphorus	0.71 ± 0.10	1.46	1.76	0.12
Potassium	4.2 ± 0.61	1.86	0.05	1.36
Sodium	1.18 ± 1.15	0.89	1.35	1.05
**Trace elements (mg/100 g)**				
Iron	32.3 ± 3.90	173.37	0.30	28.5
Copper	1.1 ± 0.10	10.22	0.06	6.1
Selenium	<5	0.05	0.07	0.007
Zinc	2.9 ± 0.30	6.37	1.19	2.0
Iodine	0.14 ± 0.00	n.r.	n.r.	n.r.

[1] *Tibbetts et al.* [32]

[2] *Tokusoglu and Ünal* [26]

[3] *United States Department of Agriculture* [29]

n.r. – not reported
